# Effect of Beta-Blocker Therapy on the Risk of Infections and Death after Acute Stroke – A Historical Cohort Study

**DOI:** 10.1371/journal.pone.0116836

**Published:** 2015-02-02

**Authors:** Ilko L. Maier, André Karch, Rafael Mikolajczyk, Mathias Bähr, Jan Liman

**Affiliations:** 1 Department of Neurology, University Medicine Göttingen, Göttingen, Germany; 2 Research group Epidemiological and Statistical Methods (ESME), Department of Epidemiology, Helmholtz Centre for Infection Research, Braunschweig, Germany; 3 German Center for Infection Research (DZIF), Braunschweig site, Braunschweig, Germany; 4 Hannover Medical School, Hannover, Germany; University of Münster, GERMANY

## Abstract

**Background:**

Infections are a frequent cause for prolonged hospitalization and increased mortality after stroke. Recent studies revealed a stroke-induced depression of the peripheral immune system associated with an increased susceptibility for infections. In a mice model for stroke, this immunosuppressive effect was reversible after beta-blocker administration. The aim of our study was to investigate the effect of beta-blocker therapy on the risk of infections and death after stroke in humans.

**Methods:**

625 consecutive patients with ischemic or hemorrhagic stroke, admitted to a university hospital stroke unit, were included in this historical cohort study. The effect of beta-blocker therapy on post-stroke pneumonia, urinary tract infections and death was investigated using multivariable Poisson and Cox regression models.

**Results:**

553 (88.3%) patients were admitted with ischemic stroke, the remaining 72 (11.7%) had a hemorrhagic stroke. Median baseline NIHSS was 8 (IQR 5–16) points. 301 (48.2%) patients received beta-blocker therapy. There was no difference in the risk of post-stroke pneumonia between patients with and without beta-blocker therapy (Rate Ratio = 1.00, 95%CI 0.77–1.30, p = 0.995). Patients with beta-blocker therapy showed a decreased risk for urinary tract infections (RR = 0.65, 95%CI 0.43–0.98, p = 0.040). 7-days mortality did not differ between groups (Hazard Ratio = 1.36, 95%CI 0.65–2.77, p = 0.425), while patients with beta-blocker therapy showed a higher 30-days mortality (HR = 1.93, 95%CI 1.20–3.10, p = 0.006).

**Conclusions:**

Beta-blocker therapy did not reduce the risk for post-stroke pneumonia, but significantly reduced the risk for urinary tract infections. Different immune mechanisms underlying both diseases might explain these findings that need to be confirmed in future studies.

## Introduction

Systemic infections are associated with poor outcome and increased mortality after ischemic and hemorrhagic stroke [1–3]. In a meta-analysis of 87 studies, the post-stroke infection rate has been estimated to be 30%, with pneumonia and urinary tract infections (UTI) accounting for the majority of cases [4].

Recent experimental studies showed an active interaction between the central nervous system and the peripheral immune system, which can result in immunosuppression and increased susceptibility for systemic infections after stroke [5–8]. This effect is thought to be a compensatory response to protect the post-ischemic brain from overwhelming and damaging inflammatory response, which is caused by infiltration of immune cells in the ischemic brain area with oxidative stress, microglial and complement activation and damage of the blood brain barrier [9, 10].

One of these immunosuppressive mechanisms after stroke is an activation of the sympathetic nervous system, resulting in an induction of anti-inflammatory phenotype immune cells [11]. In patients with ischemic and hemorrhagic stroke, increased catecholamine levels and decreased peripheral immune response have been described previously [12, 13]. Hyperactivity of the sympathetic nervous system hereby is mainly caused by lesions of the anterior medial cortex and insular cortex [14, 15]. Wong et al. investigated the mechanism of this inhibitory pathway in a mice model and detected functional changes of invariant natural killer T (iNKT) cells after medial cerebral artery occlusion (MCAO) [16]. After MCAO, the iNKT cells showed an anti-inflammatory phenotype, resulting in a high incidence of infection and increased mortality. The authors demonstrated a complete reversion of the anti-inflammatory phenotype, a complete inhibition of mortality after systemic administration of the nonspecific beta-blocker propranolol and a 50% reduction of mortality after chemical depletion of peripheral neuronal terminals containing noradrenaline 24 h after MCAO. These data suggest a post-ischemic, noradrenergic immunosuppressive pathway and a central role of iNKT cells in the systemic immune reaction after stroke. However, it remains unclear, which receptors are involved in mediating this immunosuppressive effect and if the inhibition of immunosuppression after administration of propranolol is caused by β_1_- or β_2_-antagonism.

The aim of our study was to investigate the effect of commonly prescribed beta-blockers on the incidence of post-stroke infections and mortality. We hypothesized, that patients with beta-blocker therapy are less likely to develop post-stroke infection and have a decreased mortality compared to patients without beta-blocker therapy.

## Materials and Methods

### Patient population and clinical characteristics

In this historical cohort study, we evaluated clinical data from 625 consecutive patients with clinically and radiologically diagnosed acute ischemic or hemorrhagic stroke, who were admitted to the stroke unit or neurological intensive care unit at the University Medicine Göttingen, Germany between 2011 and 2013. Inclusion criteria were an acute major ischemic stroke located in the medial cerebral artery (MCA) territory or lobar/non-lobar, supratentorial hemorrhagic stroke (National Institute of Heath Stroke Scale (NIHSS) ≥ 4). Exclusion criteria were evidence for systemic infection (clinically evident and confirmed by routine diagnostic procedures) or malignancy (dependent on the patients past medical history) at baseline and immunosuppressive therapy prior to hospitalization. Patients with subarachnoidal bleeding, pons or cerebellar hemorrhages, ischemic stroke in other territories than MCA, transient ischemic attacks or NIHSS ≤ 4 at baseline were excluded.

Exposure of interest was beta-blocker therapy. Patients receiving beta-blocker therapy prior to stroke and continued the beta-blocker therapy during their in-patient stay were considered as beta-blocker exposed. Patients without beta-blocker therapy before and during the follow-up period were considered as non-exposed. Patients with discontinuation of beta-blocker therapy during the follow-up period were not considered for inclusion in this study.

Pneumonia within seven days after stroke was defined a-priori as the primary outcome of interest. Under the assumption of a baseline pneumonia rate of 30% and about 50% exposure to beta-blockers, 625 patients were required to show a difference of 10% in pneumonia rates between groups with 80% power (two-sided alpha = 0.05). UTI within seven days after stroke and death were considered as key secondary outcomes.

The diagnosis of pneumonia was made, if new or persistent infiltrations on chest x-ray in combination with leukocytosis with left shift or leukocytopenia, at least 50% increase of C-reactive protein (CRP) or procalcitonin compared to baseline, hypo- (<36.5°C) or hyperthermia (>38.5°C), cough with purulent sputum or characteristically rattling noises on auscultation were evident. The diagnosis of UTI was based on clinical symptoms in combination with findings on dipstick (nitrite and leucocyte positive), which was confirmed by the presence of growth of >10^5^ organisms per mL. Risk of death was investigated using mortality rates within 7 and 30 days after stroke. Additional secondary outcomes were changes in leukocyte count, CRP level, NIHSS, follow-up modified ranking scale (mRS) and Barthel-index.

Data collection has been performed on the basis of a standardized protocol using electronic chart review and reports from neurological rehabilitation centers.

Ethics approval was sought from the ethics committee of the University Medical Center Göttingen which confirmed no need for approval for this study due to its retrospective nature and the fact that it is based on routinely collected anonymized data only (Reg.-nr. 1/5/13An).

### Statistical analysis

Statistical analyses were performed using SPSS 20 (IBM SPSS Statistics, Armonk, NY, USA) and Stata 11 (StataCorp, College Station, US). Baseline characteristics of all patients are shown as percentage or mean ± standard deviation (SD), if normally distributed, and as median + interquartile range (IQR), if not. Multivariable Poisson (for post-stroke infections) and Cox regression models (for risk of death) were used to investigate the effects of beta-blocker therapy. Due to the complex network of potential confounders and the high potential for multicollinearity between the variables beta-blocker therapy, atrial fibrillation and hypertension, directed acyclic graphs (DAG) approach was applied using DAGitty [17]. This was done in addition to a standard statistical approach for model building (confounder selection based on changes in the point estimate of the exposure of interest) in order to derive a minimal sufficient adjustment set of confounders that was not affected by multicollinearity. Under the assumptions made for the DAG approach (based on literature review) age, sex and stroke severity (estimated by baseline NIHSS) were selected as the primary adjustment set of choice. While hypercholesterolemia and statin therapy were not considered as confounders of an association between beta-blocker therapy and post-stroke infections, a-priori evidence suggested that statin therapy might be an effect modifier of this association [18, 19]. We investigated this potential effect modification and present a p-value for interaction based on a Likelihood Ratio Test.

In order to account for a competing risk situation between death and infections, sensitivity analyses were performed, in which patients who died within the first seven days after stroke were considered to have developed the post-stroke infections.

ANCOVAs were used to evaluate the exposure effect on continuous secondary outcomes. Changes in outcome parameters were calculated as baseline-values minus follow-up value (for NIHSS) or highest value (for CRP and leukocyte count). P-values ≤ 0.05 were considered to be statistically significant.

## Results

### Baseline characteristics of the study population

625 patients were included in this study, of which 552 (88.3%) had an acute ischemic stroke and 73 (11.7%) a hemorrhagic stroke. Most frequently prescribed beta-blockers were metoprolol (145 patients, 48.2%) and bisoprolol (122 patients, 40.5%).

Patients with beta-blocker therapy were significantly older (p = 0.001), more likely to be female (p = 0.003) and had more co-morbidities (diabetes mellitus (p<0.001), hypertension (p<0.001), atrial fibrillation (p<0.001)) than those without beta-blocker therapy (Table 1).

**Table 1:** Baseline characteristics of patients with and without beta-blocker therapy (n = 625).

	Beta-Blocker (n = 301)	No-Beta-Blocker (n = 324)	P-value*
Age (mean ± SD)	75 ± 11	72 ± 13	0.001
Sex (n, % male)	144 (47.8)	193 (59.6)	0.003
Ischemic stroke (n, %)	268 (89)	284 (87.7)	0.591
Hemorrhagic stroke (n, %)	33 (11)	40 (12.3)	0.591
Diabetes mellitus (n, %)	107 (35.5)	69 (21.3)	<0.001
Arterial hypertension (n, %)	288 (95.7)	246 (75.9)	<0.001
Atrial fibrillation (n, %)	136 (45.2)	77 (23.8)	<0.001
Follow up time (days, IQR)	15, 8–37	19, 9–40	0.269
Statin therapy (n, %)	216 (71.8)	226 (69.8)	0.582

SD: Standard deviation, IQR: Interquartile range,

*t-tests, Wilcoxon rank-sum tests and chi-square tests as appropriate

### Effect of beta-blocker therapy on primary and secondary key outcomes

**Figure 1:**
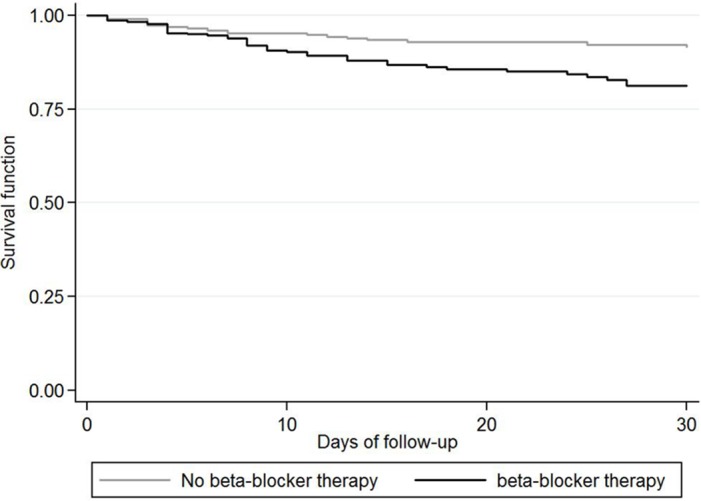
Kaplan-Meier plot displaying survival after stroke in patients with and without beta-blocker therapy. Patients with beta blocker therapy showed a higher 30 days mortality than those without beta blocker therapy in the univariable (log-rank test, p = 0.003) and multivariable analyses (Cox regression model, p = 0.006).

There was no difference for the risk of post-stroke pneumonia between patients with and without beta-blocker therapy (Rate Ratio (RR) = 1.00, 95% CI 0.77–1.30, 0.995). UTI rates were considerably reduced in the beta-blocker group compared to patients without beta-blocker therapy (RR = 0.65, 95% CI 0.43–0.98, p = 0.040). Sensitivity analyses assuming that all patients who died within the first seven days after stroke would have developed an infection still showed a risk reduction for UTI (RR = 0.80, 95%CI 0.56–1.15, p = 0.233) and maintained the absence of a difference for post-stroke pneumonia (Table 2). All-cause mortality 7 days after stroke did not differ between groups (Hazard Ratio = 1.36, 95% CI 0.65–2.77, p = 0.425), while all-cause mortality after 30 days was substantially higher among patients treated with beta-blockers (HR = 1.93, 95% CI 1.20–3.31, p = 0.006) (Fig. 1). There was no difference for the risk of myocardial infarction between patients with and without beta-blocker therapy (HR = 0.78, 95% CI 0.41–1.49, p = 0.458).

**Table 2:** Post-stroke pneumonia, urinary tract infection and mortality in patients with and without beta-blocker therapy (n = 625).

	Beta-Blocker (n = 301)		No-Beta-Blocker (n = 324)		HR/RR	95% CI	
Main analysis	Events	follow-up days	Events	follow-up days			
Post-stroke pneumonia (7 days after stroke)	114	1960	123	2123	1.00	0.77–1.30	0.995
Urinary tract infection (7 days after stroke)	40	1960	57	2123	0.65	0.43–0.98	0.040
Death (7 days after stroke)	18	1960	15	2123	1.36	0.65–2.77	0.425
Death (30 days after stroke)	42	5418	22	6168	1.93	1.20–3.10	0.006
Sensitivity analysis‡							
Post-stroke pneumonia (7 days after stroke)	126	1960	123	2123	1.07	0.83–1.37	0.612
Urinary tract infection (7 days after stroke)	56	1960	68	2123	0.80	0.56–1.15	0.233

HR/RR: Hazard-Ratio and Rate Ratio (adjusted for age, sex and baseline NIHSS) obtained using Poisson (pneumonia, urinary tract infection) and Cox (death) regression models, CI: Confidence interval,

*Likelihood Ratio Test,

‡ competing risk situation

Sensitivity analyses assessing the specific effects of metoprolol or bisoprolol therapy showed similar point estimates as the main analyses (data not shown).

### Effect modification by statin therapy

The effect of beta-blocker therapy on UTI rates was considerably higher in patients without statin therapy (RR = 0.33, 95% CI 0.15–0.75) compared to patients with statin therapy (RR = 0.86, 95% CI 0.53–1.41, p-value for interaction = 0.043, Table 3). There was a trend for a similar effect modification regarding post-stroke pneumonia, although it was not statistically significant (p = 0.220, Table 3).

**Table 3:** Effect of beta-blocker therapy on post-stroke infections stratified by statin therapy.

	Effect of beta-blocker therapy				
	Urinary tract infection			Post-stroke pneumonia	
Statin therapy (n = 442)	0.86 (0.53–1.41)			1.19 (0.85–1.67)	
No statin therapy (n = 183)	0.33 (0.15–0.75)	p = 0.043*		0.87 (0.58–1.32)	p = 0.220*

*p-value for interaction based on a Likelihood Ratio Test comparing a model with interaction term with the corresponding model without interaction term

### Effect of beta blocker therapy on additional secondary outcomes

Patients with beta-blocker therapy showed a trend to lower NIHSS values at baseline (p = 0.052), while there was no difference in change in NIHSS, CRP or leukocyte count between both groups (Table 4). mRS and Barthel-index were available for 203 patients with a median follow-up of 50 (IQR, 37–73) days. There was no difference in mRS or Barthel-index between patients with and without beta-blocker therapy.

**Table 4:** C-reactive protein, leukocyte count, NIHSS, modified ranking scale and Barthel-index in patients with and without beta-blocker therapy (n = 625).

	Beta-Blocker (n = 301)	No-Beta-Blocker (n = 324)	P-value*
Baseline NIHSS (Median, IQR)	8, 5–15	9, 6–16	0.052
Change NIHSS (score ± SE)	1.0 ± 0.4	1.5 ± 0.3	0.289
Change CRP (mg/l ± SE)	342.1 ± 46.1	426.3 ± 42.4	0.089
Change Leukocyte count (*10^3/μl ± SE)	2.5 ± 0.5	3.1 ± 0.4	0.270
mRS follow-up (n = 203; score ± SE)	2.8 ± 0.1	2.8 ± 0.1	0.914
Barthel-index follow-up (n = 203; score ± SE)	59.2 ± 4.5	61.4 ± 4.3	0.661

NIHSS: National Institute of Health Stroke Scale; CRP: C-reactive protein; mRS: modified Ranking Scale; SE: Standard error; ANCOVAs adjusted for age, sex, baseline NIHSS and individual follow-up; Change in outcome parameters were calculated as follows: baseline value—follow-up value (for NIHSS) or highest value—baseline value (for CRP and leukocyte count)

## Discussion

In the present study, we investigated the effect of beta-blocker therapy on infectious complications after stroke. Contrary to our hypotheses, beta-blocker therapy was not associated with a lower risk for post-stroke pneumonia. However, we found a significant risk reduction of 35% for UTIs in patients treated with beta-blockers. There was no difference in mortality after 7 days, while we found a higher mortality in patients receiving beta-blocker therapy after 30 days.

There is growing evidence that various mechanisms lead to high susceptibility for infections after stroke. One of these mechanisms is the activation of the sympathetic nervous system, which leads to peripheral immunosuppressive effects [11]. These effects have been shown to be reversed by propranolol administration indicating a noradrenergic pathway and involvement of β_1_- or β_2_-receptors [16]. Based on this evidence, we expected a decrease in infectious complications after stroke among patients receiving beta-blockers. The observed difference in the effect of beta-blocker therapy on pneumonia and UTI might be attributable to different mechanisms by which infection and inflammation are mediated in both conditions. Pneumonia after stroke is mostly related to aspiration. Initial inflammation is triggered by chemicals like gastric acid, resulting in a pneumonitis. A second step is a bacterial super-infection resulting in pneumonia. In contrast, UTIs are primarily caused by infectious agents and are often catheter-associated. iNKT cells have been described to play a central role in the immune response after stroke and are known to recognize bacterial glycolipids and endogenous moieties that can function as alarmins [16, 20]. Given the different mechanism in post-stroke pneumonia and UTI, iNKT cells might be differently involved in immune response in both conditions. Activation of iNKT in the initial phase of UTI might therefore be more striking through both the presentation of bacterial antigens and recognition of circulating alarmins. Therefore, the influence of beta-blocker therapy on rates of UTIs might be explained by the different involvement and extent of activation of iNKT cells. However, it is also possible that both conditions could involve other subsets of immune cells, initially reacting on chemical or infectious agents, which are being influenced differently by beta-blocker therapy. Moreover, UTIs are usually caused by gram-negative bacteria, while pneumonia shows a more complex picture with many classic gram-positive germs involved. Immune reaction to both groups is quite distinct so that it might be possible that the protective effect of beta-blockers is unique to gram-negative bacteria.

In other studies lower mortality, immunomodulatory effects and enhanced inflammatory potential of immune cells have been found for patients with sepsis and beta-blocker therapy [20–23]. Ackland et al. found protective effects in rats allocated with β_1_-antagonists before a septic insult, resulting in a significant reduction of mortality by preventing an overwhelming pro-inflammatory state [24]. This study indicates that β_1_-receptors are involved in peripheral immune modulation of systemic inflammatory processes and might influence regulating processes to guarantee an effective immune response.

Studies focusing on the role of beta-blocker therapy after stroke found conflicting results on mortality and stroke outcome. In the beta-blocker stroke (“BEST”) trial, patients continuing their prior therapy with propranolol or atenolol had a better outcome after stroke [25]. However, the authors also found a higher mortality in patients taking beta-blockers, which was associated with higher co-morbidities in the beta-blocker group, while infection rates were not reported.

In view of the findings of beneficial effects of beta-blocker therapy in patients with sepsis and the conflicting results in patients with stroke, the interpretation of the higher medium-term risk for death of patients with beta-blocker therapy in our study is difficult. Patients in the beta-blocker group had significantly more co-morbidities and were at higher risk for cardiovascular disease. Therefore, overall risk of death could be higher in these patients and full adjustment of confounding factors might not have been possible. The influence of cardiovascular disease on mortality after stroke can be underlined by findings from a study by Dziedzic et al., who found a lower 30-day mortality of patients with beta-blocker therapy and stroke, which was no longer statistically significant after removing patients from the analysis, who died because of cardiac complications [26]. However, as death of any cause was not a primary outcome of this study, we did not take into account potential confounders of the association of beta-blockers and death which were not affecting the link between beta-blockers and infection. This is supported by the fact that effects of post-stroke infections and death are heading in opposite directions and that beta-blocker therapy did not affect mortality after seven days. Moreover, rates of myocardial infarction did not differ between patients with and without beta-blocker therapy in our study.

Besides the causes for higher mortality rates in the beta-blocker group, we had to consider the differences in mortality rates between groups due to a potential competing risk situation of the outcome death and other endpoints. Patients who died earlier might have been more likely to develop infectious complications if they had not died. In our sensitivity analyses using the most extreme scenario (i.e. that all people who died would have developed the respective infection) 1/3 of the observed effect of beta-blocker therapy on UTIs was removed leading to a point estimate of 0.80. Thereby, even this extremely conservative scenario confirmed a clinically relevant risk reduction for UTIs in patients with beta-blocker therapy.

In a study by Laowattana et al, beta-blocker therapy was associated with lower stroke severity at baseline [27]. We support this evidence by showing a trend towards a lower baseline NIHSS in the beta-blocker group. However, it seems unlikely that the lower NIHSS by one point (median difference) in the beta-blocker group explains the lower rate of UTIs in the same group through lower stroke severity. In addition, there was no difference in change of NIHSS between the groups over time and therefore no indication that this could increase the risk to develop an infection in one of the groups.

In the same study, thrombin and erythrocyte sedimentation rate were lower in patients taking beta-blockers indicating less systemic inflammation. In contrast, in our study beta-blocker therapy was not associated with less pronounced changes in leukocyte count or CRP, both indicators for systemic infections. These findings suggest different effects of beta-blocker therapy on specific markers of systemic infections, indicating that these markers are not influenced equally by increased catecholamine-levels.

Although β_1_-receptors seem to be involved in immunomodulating effects after stroke, either indirectly by lowering the sympathetic tone or via direct antagonism influencing the function of peripheral immune cells, there is evidence for β_2_-receptor-mediated immunosuppressive mechanisms associated with an increased sympathetic tone. In an in vitro study by Platzer et al., allocation of catecholamines induced a β_2_-receptor-mediated production of the immunosuppressive cytokine IL-10 in monocytic cells [28]. Unselective beta-blockers could therefore combine β_1_- and β_2_-receptor associated inhibition of immunosuppression after stroke. It seems to be important to compare the incidence of infections after stroke in patients receiving β_1_-selective beta-blockers with the incidence in patients receiving non-selective beta-blockers and no beta-blockers. However, studying this question is difficult because indications for unselective beta-blockers are limited due to side effects like bronchospasm and hypoglycemia.

Recent studies found pleiotropic effects of 3-hydroxy-3-methyl-glutaryl coenzyme A (HMG-CoA) reductase inhibitors (statins), frequently prescribed after stroke to lower serum cholesterol, on endothelial function, cell proliferation, inflammatory response, immunological reactions and platelet function [19]. Statins have been shown to be involved in immunological pathways, involving the function of immune response on multiple levels (gene transcription factors, cytokines, chemokines, immune cell function and proliferation) [29]. Other studies also showed anti-inflammatory effects of statins in autoimmune diseases. Therefore, we performed a stratified analysis in order to elucidate a potential modifying effect of statin therapy on the effect of beta-blocker therapy on post-stroke infections. The effect of beta-blocker therapy on UTI rates was significantly larger in patients without statin therapy. This might be explained by an anti-proliferative effect of statins on T-cells [30]. Moreover, in a study by Fehr et al., statin administration was associated with major histocompatibility class II (MHC II) antigen down-regulation and the inhibition of superantigen-mediated T-cell activation [31]. In the context of the findings of Wong et al [16], these effects of statins on T-cell function might explain, why beta-blocker associated reduction of infections after stroke were predominantly found in patients without statin therapy. In patients with statin therapy, the effect of beta-blockers is likely to be lower because of an anti-proliferative effect of statins on iNKT cells, which have been demonstrated to play a central role in stroke induced peripheral immunosuppression.

A major strength of our study is the inclusion of a high number of consecutive stroke patients in a university medical center. There are several limitations of our study; namely, the monocentric retrospective study design, as well as a lack of information about urinary catheters, dysphagia and the use of nasogastric tubes and cardiac co-morbidities. However, it is likely, that most of the included patients at least temporarily had a nasogastric tube and urinary catheter, because we included patients with major ischemic or hemorrhagic stroke (median (IQR) NIHSS in the beta-blocker group 8 (5–15), in the no-beta-blocker group 9 (6–16)), who had severe neurological deficits and were bed bound in the acute phase after stroke. Moreover, with respect to the analyses performed in our study we do not think that urinary catheter rates or naso-gastric tubes could have a major confounding effect on the association between beta-blocker therapy and post-stroke infection rates since decision on a urinary catheter or naso-gastric tube is not dependent on beta-blocker therapy itself.

Further prospective and randomized studies are necessary to investigate the role of beta-blocker therapy on the incidence of infections after stroke. Future research might result in the development of immunomodulatory therapies for acute stroke that can help preventing stroke-associated infections and lowering stroke case fatality rates.
