# Correction: Effect of Beta-Blocker Therapy on the Risk of Infections and Death after Acute Stroke - A Historical Cohort Study

**DOI:** 10.1371/journal.pone.0121940

**Published:** 2015-03-26

**Authors:** The PLOS ONE Staff

The first two authors, Ilko Maier and André Karch, should be noted as joint senior authors of this work. The last three authors, Rafael Mikolajczyk, Mathias Bähr, and Jan Liman, should be noted as joint secondary authors of this work.
